# Discovery of EST-SSRs in Lung Cancer: Tagged ESTs with SSRs Lead to Differential Amino Acid and Protein Expression Patterns in Cancerous Tissues

**DOI:** 10.1371/journal.pone.0027118

**Published:** 2011-11-04

**Authors:** Mohammad Reza Bakhtiarizadeh, Mansour Ebrahimi, Esmaeil Ebrahimie

**Affiliations:** 1 Department of Animal Science, University of Tehran, Karaj, Iran; 2 Department of Biology & Bioinformatics Research Group, University of Qom, Qom, Iran; 3 School of Molecular and Biomedical Science, The University of Adelaide, Adelaide, Australia; Florida International University, United States of America

## Abstract

Tandem repeats are found in both coding and non-coding sequences of higher organisms. These sequences can be used in cancer genetics and diagnosis to unravel the genetic basis of tumor formation and progression. In this study, a possible relationship between SSR distributions and lung cancer was studied by comparative analysis of EST-SSRs in normal and lung cancerous tissues. While the EST-SSR distribution was similar between tumorous tissues, this distribution was different between normal and tumorous tissues. Trinucleotides tandem repeats were highly different; the number of trinucleotides in ESTs of lung cancer was 3 times higher than normal tissue. Significant negative correlation between normal and cancerous tissue showed that cancerous tissue generates different types of trinucleotides. GGC and CGC were the more frequent expressed trinucleotides in cancerous tissue, but these SSRs were not expressed in normal tissue. Similar to the EST level, the expression pattern of EST-SSRs-derived amino acids was significantly different between normal and cancerous tissues. Arg, Pro, Ser, Gly, and Lys were the most abundant amino acids in cancerous tissues, and Leu, Cys, Phe, and His were significantly more abundant in normal tissues than in cancerous tissues. Next, the putative functions of triplet SSR-containing genes were analyzed. In cancerous tissue, EST-SSRs produce different types of proteins. Chromodomain helicase DNA binding proteins were one of the major protein products of EST-SSRs in the cancerous library, while these proteins were not produced from EST-SSRs in normal tissue. For the first time, the findings of this study confirmed that EST-SSRs in normal lung tissues are different than in unhealthy tissues, and tagged ESTs with SSRs cause remarkable differences in amino acid and protein expression patterns in cancerous tissue. We suggest that EST-SSRs and EST-SSRs differentially expressed in cancerous tissue may be suitable candidate markers for lung cancer diagnosis and prediction.

## Introduction

Rapid generation of genomics and functional genomics data has provided novel, fast, and inexpensive tools in functional dissecting of vital phenomena like cancer identification and prediction. Expressed sequence tags (ESTs) are sequenced from parts of the coding regions of the genome under certain biological conditions [1]. ESTs can be developed from cDNA libraries to obtain expression information in contrasting environmental conditions or across developmental stages to provide an inexpensive source of gene-based DNA markers [2]. Collections of ESTs have been generated in different human tissues, which provides a unique opportunity for searching for SSR motifs and developing the corresponding microsatellite markers [3]. In recent years, the increasing number of deposited ESTs in data banks has accelerated research in this field. A vast amount of deposited EST sequences in Harvard University (The Gene index Project, http://compbio.dfci.harvard.edu/tgi/tgipage.html) and NCBI) http://www.ncbi.nlm.nih.gov/blast) provides the opportunity of precise consideration of different biological events by EST-SSR analysis not only in DNA level but also in amino acid and functional protein level.

The length of microsatellites or SSRs varies from one to six (or more) units of tandem-repeated sequences. These sequences are ubiquitously distributed in prokaryotic and eukaryotic genomes and can be found in both the coding and non-coding sequences of higher organisms [4], [5], [6], [7]. In comparison with other molecular markers, SSRs are uniquely characterized by their simplicity, abundance, ubiquity, variation, co-dominance, and multi-alleles nature among genomes [8]. Due to the potential of abundant polymorphisms, SSRs have become a valuable source of genetic markers and have been broadly applied to various areas of genetic research, including genome variation, establishment of genetic maps, integration of physical and genetic maps, determination of evolutionary relationships, and comparative genome analyses [8], [9], [10].

EST-SSRs, which are a combination of EST and SSRs, offer several advantages over the other genomic DNA-based markers; these advantages include being able to detect the variation in the expressed portion of the genome and having a higher level of transferability to closely related species than do genomic SSR markers [11] [12]. There is some evidence of lower EST-SSR variation in comparison with the introns or intergenic regions, but even the lowest estimates suggest that at least 25% of EST-SSRs are polymorphic [12]. Regarding the existence of EST-SSRs in transcribed regions of the genome, these sequences can lead to the development of gene-based maps for identifying functional candidate genes and increasing the efficiency of marker-assisted selection.

In contrast to primary assumption which suggests SSRs are not functional elements, new studies have demonstrated that the genomic distribution of SSRs is nonrandom, presumably because of their effects on chromatin organization, regulation of gene activity, recombination, DNA replication, cell cycle, and mismatch repair) [13]. Subsets of SSRs, namely, trinucleotide repeats, are of great interest because of their role in many human neurodegenerative disorders and cancers [14], [15]. In fact, the expansion of triplet repeats is responsible for the abovementioned genetic diseases in which the rate of mutation and, consequently, disease induction depends on the number of tandem units within the repeat [14], [15]. For example, the role of (CAG)_n_ repeat expansions in spinobulbar muscular atrophy and (CTG)_n_ repeat expansions in myotonic dystrophy are well documented [16]. Originally, expansions were limited to trinucleotide repeats, but it is now clear that tetrameric, pentameric and even dodecameric repeats can also expand and lead to human disease [17]. Microsatellite instability reflects replication errors induced by a defective function of mismatch repair genes, resulting in appearance of novel, non-inherited alleles in tumor cells. As a result, co-expressed SSRs with ESTs can be correlated with clinicopathological features of cancer, its formation, and tumor development. SSRs have been used in cancer genetics and indirect cancer diagnosis to help unravel the genetic basis of tumor formation and cancer progression. Microsatellite instability has been reported in colorectal cancer [18], breast cancer [3], [19], ovarian cancer, and other cancers [20], [21], [22], [23], [24], [25], [26]. However, to date, there has been no report on the application of EST-SSRs in lung cancer.

Lung cancer is the leading cause of cancer-related deaths in both women [27] and men [28] throughout the world; it has surpassed breast cancer as the leading cause of cancer deaths in women [27]. For the first time, we report differential behavior of EST-SSRs between normal and cancerous lung tissues in DNA, amino acid, and annotated-protein levels. At first, distribution frequency and type of EST-SSRs were compared in normal and cancerous lung tissues. Then, EST-derived amino acid frequencies were compared between normal and cancerous tissues. Finally, the putative functions of SSR-containing genes were analyzed in normal and cancerous tissues. The result of this study can be used for developing ESS-SSR-based detection tool for lung cancer in future studies and opens a new avenue in investigating differential expression of EST-SSRs as one of the possible causes of lung cancer.

## Methods

### Retrieval of EST libraries

EST libraries of lung (1 from normal and 2 from cancerous tissues) were downloaded from the EST collection (The Gene Index Project) of Harvard University (http://compbio.dfci.harvard.edu/tgi/tgipage.html). The EST library from normal lung tissue (Cat No. LB36) contains 11652 ESTs. Two EST libraries from malignant lung tumors were used: an undifferentiated large-cell carcinoma library containing 6556 ESTs (Cat No. #5F8) and a poorly differentiated squamous-cell carcinoma containing 6662 ESTs (Cat No. LF43).

Large cell carcinomas are a group of cancers with large, abnormal-looking cells. These tumors usually begin along the outer edges of the lungs. Squamous cell carcinoma, also called epidermoid carcinoma, is the most common type of lung cancer in men. It often begins in the bronchi, and usually does not spread as quickly as other types of lung cancer (http://www.umm.edu/respiratory/lungcan.htm).

### Comparison of EST-SSRs between the normal and cancerous libraries

EST-SSRs were identified by SSR Locator software as described previously [29]. SSR Locator is a tool for detecting and characterizing micro-and minisatellites in DNA sequences. Beyond finding the repeating sequences, the program can also design primers, simulate PCR reactions, and make global alignments between homologous regions obtained from the PCR simulation.

To evaluate the EST-SSR distribution pattern between normal and cancerous tissues, the EST sequences of two cancerous libraries were first pooled. Next, the EST sequences of normal and cancerous libraries were scanned for SSR motifs ranging in length from 2 to 6 nucleotides with dinucleotide repeat numbers ≥7, trinucleotide repeat numbers ≥6, tetranucleotide repeat numbers ≥5, pentanucleotide repeat numbers ≥5, and hexanucleotide repeat numbers ≥5.

### Comparison of EST-SSRs between cancerous libraries

Two EST libraries from cancerous tissues (Cat No. #5F8 and Cat No. LF43) were used to test whether the EST-SSR distribution is similar between cancerous tissues. The EST sequences from the cancerous libraries were searched with SSR Locator for SSRs motifs ranging from 2 to 6 nucleotides in length. The repeat number parameters were as follows: ≥7 for dinucleotides, ≥6 for trinucleotides, ≥5 for tetranucleotides, ≥5 for pentanucleotides, and ≥5 for hexanucleotides.

### Primer designing for EST-SSRs

For each microsatellite-containing EST, primers were designed using Primer3 (http://frodo.wi.mit.edu/primer3/) by running the software in a batch mode with the assistance of the SSR locator interface module. The primer design function was used to determine if the sequences had sufficient flanking sequences for designing primers. The major parameters for primer design were as follows: PCR product size 100–300 bp, primer length 18–25 bp with 20 bp as the optimum, optimum annealing temperature 58–63 °C with 60 °C as the optimum, and a minimum GC content of 30%, with 50% being the optimum.

### Amino acid distributions of ESTs with trinucleotide repeats

The type of amino acids and their distributions in normal and cancerous tissues were predicted for ESTs with trinucleotide repeats using SSR Locator software. Translating the EST-SSRs to their corresponding amino acids provides some clues about the differences and similarities between normal and cancerous tissues at the protein level.

### Statistical analysis

To understand the similarities and differences between cancerous and normal lung tissues in generation of EST-SSRs, the Paired T-test was employed to compare the number of expressed SSRs in each class of EST-SSRs (dinucleotides, trinucleotides, tetranucleotides, pentanucleotides, and hexanucleotides) between the normal and cancerous libraries. Different sequences of tandem repeats in each class were used as pairing clusters.

In addition, to evaluate the co-linearity of cancerous and normal tissues in generating different EST-SSRs, Pearson correlations were calculated using MINITAB14 software (www.minitab.com).

After predicting the amino acid composition of ESTs containing trinucleotide tandem repeats, the number of amino acid loci and number of amino acid repeats were compared between lung cancerous and normal tissues by Paired T-test. Different types of amino acids were assumed as pairing clusters.

In addition, the expressed SSRs in each class of EST-SSRs were compared by the Paired T-test between cancerous libraries to examine their distribution in cancerous tissues.

### Annotation of SSR-containing sequences

To shed light on the putative functions of SSR-containing genes in cancerous and normal tissues, Fasta files of all identified EST-SSRs in cancerous and normal lung tissues were subjected to Blast2GO (http://www.blast2go.org/) software and were run against the non-redundant (nr) protein database of the NCBI) http://www.ncbi.nlm.nih.gov/blast); the obtained hits were compiled [30]. EST-SSRs with a best e-value of 10^−6^ or lower were assigned a putative identity.

## Results

### Frequency and distribution of EST-SSRs in normal and cancerous tissues

In total, 24870 ESTs were analyzed by SSR locator; 13218 ESTs belonged to lung cancerous tissue, and 11652 ESTs belonged to normal tissue (Table 1). Analyzing a large number of ESTs in both cancerous and normal tissues provided an opportunity to monitor precisely the behavior of the expressed SSRs in lung cancer. The average lengths of the EST sequences in cancerous and normal tissues were 478 and 288 bp, respectively. This length difference clearly shows that when a lung tissue commences to generate a tumor, a shift in alternative splicing occurs in the whole genome, thereby producing longer ESTs and proteins. Further studies on alternative splicing using the abovementioned EST libraries may unravel the splicing modulation throughout the genome in lung cancer. As presented in Table 1, in cancerous tissue, 238 SSRs were found on 227 ESTs sequences. In contrast, 208 SSR were identified on 184 ESTs were found in normal tissue (Table 1).

**Table 1:** A summary of the ESTs and EST-SSRs distribution in cancerous and normal lung tissues.

ESTs and EST-SSRs parameters	Cancer tissue	Normal tissue
Total number of ESTs	13218	11652
Average length of EST sequences	478 bp	288 bp
Total number of identified SSRs	238	208
Total number of SSR-derived ESTs (Number of ESTs containing SSR)	227	184
Number and frequency of EST sequences containing one SSRs	217 (95.59%)	160 (86.95%)
Number and frequency of EST sequences containing two SSRs	9 (3.96%)	24 (13.04%)
Number and frequency of EST sequences containing three SSRs	1 (0.44%)	0 (0.00%)

**Figure 1:**
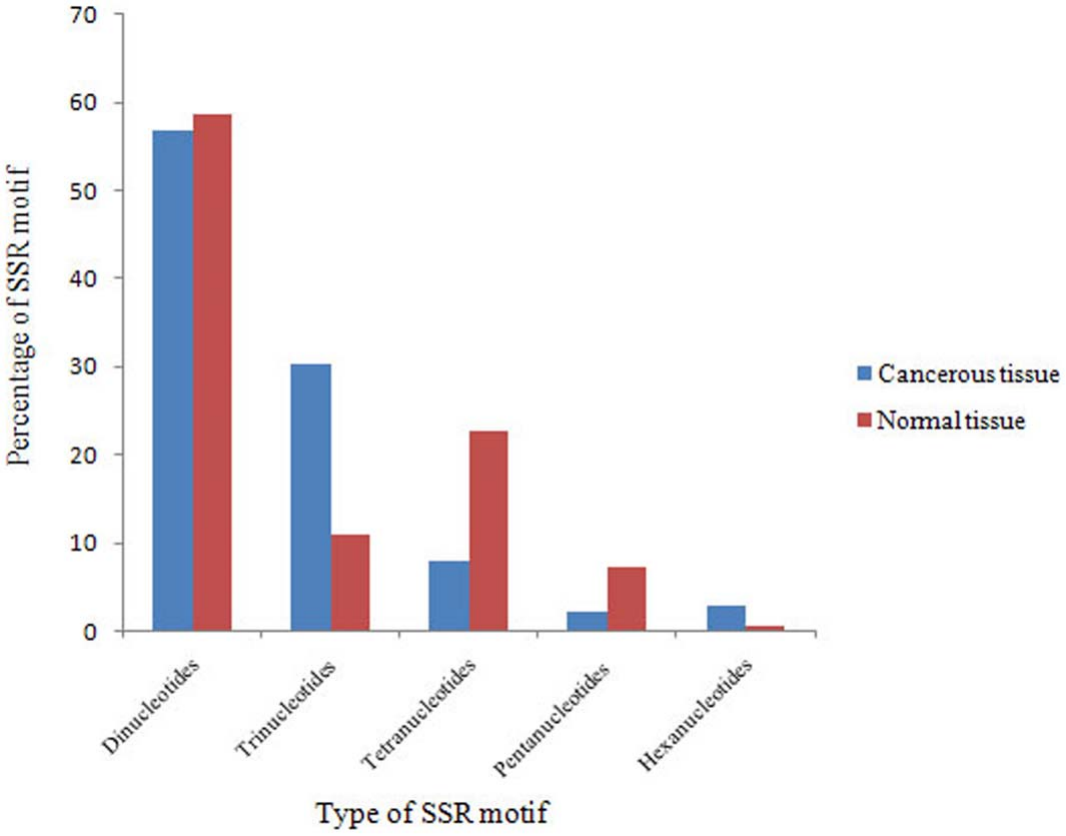
The distribution of EST-SSRs in normal and cancerous lung tissues. The percentages of dinucleotides, trinucleotides, tetranucleotides, pentanucleotides, and hexanucleotides have been compared between cancerous and normal tissues. Trinucleotide SSR repeats are abundant in the ESTs of lung cancerous tissue.

In normal tissue, the percentages of dinucleotides, trinucleotides, tetranucleotides, pentanucleotides, and hexanucleotides were 58.65%, 11.05%, 22.59%, 7.21%, and 0.48%, respectively (Figure 1). In contrast, in cancerous tissues, 56.72%, 30.25%, 7.98%, 2.10%, and 2.94% of total SSRs were assigned to dinucleotides, trinucleotides, tetranucleotides, pentanucleotides, and hexanucleotides, respectively (Figure 1).

Based on Figure 1, the greatest difference between normal and cancerous tissues belongs to trinucleotides. In other words, trinucleotides are more abundant in the cancerous library than the normal library (30.25% versus 11.05%, respectively). This finding confirms previous results on neurodegenerative disorders and other cancers in which triplet repeats are expanded in tumor tissue [14], [15]. According to Bacolla et al. (2008), there is a positive correlation between the rate of defective mutations and disease induction with an expansion of triplet repeats. Another question still remains: is there any difference between the types of expressed SSRs in each class of repeat units?

### Comparative analysis of EST-SSR types between normal and cancerous lung tissues

**Figure 2:**
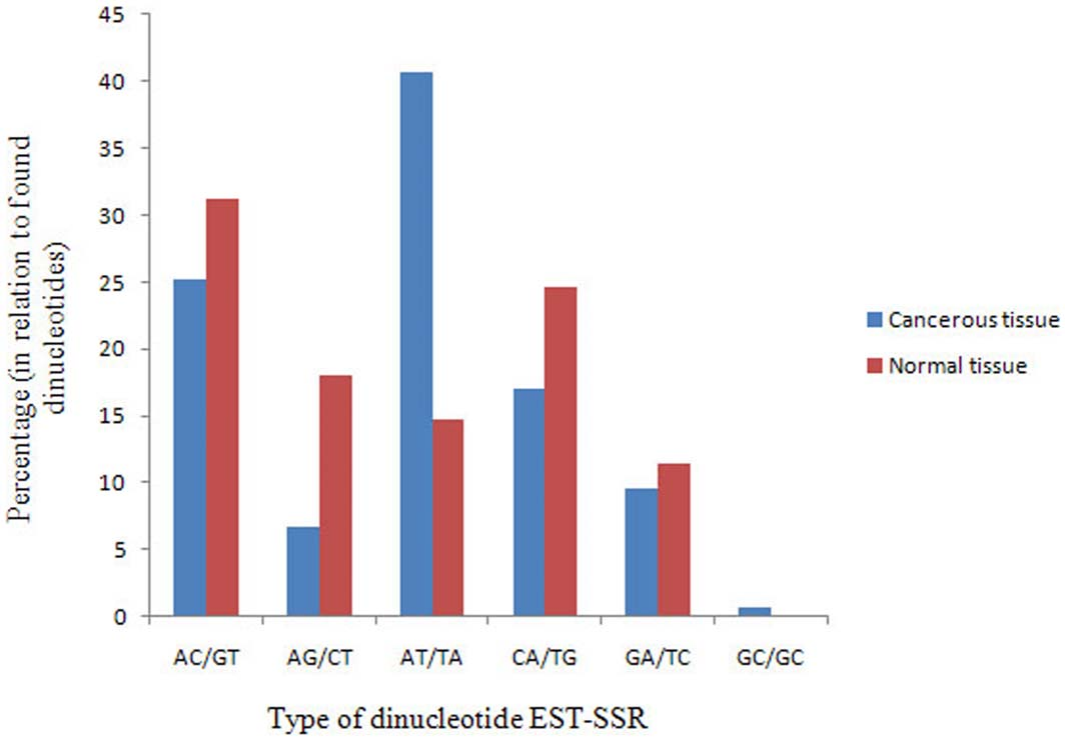
The distribution of different sequences of expressed dinucleotide SSRs in normal and cancerous lung tissues. AT/TA was more frequent in cancerous tissue than in normal tissue. In addition, GC/GC was detected solely in cancerous tissue.

**Figure 3:**
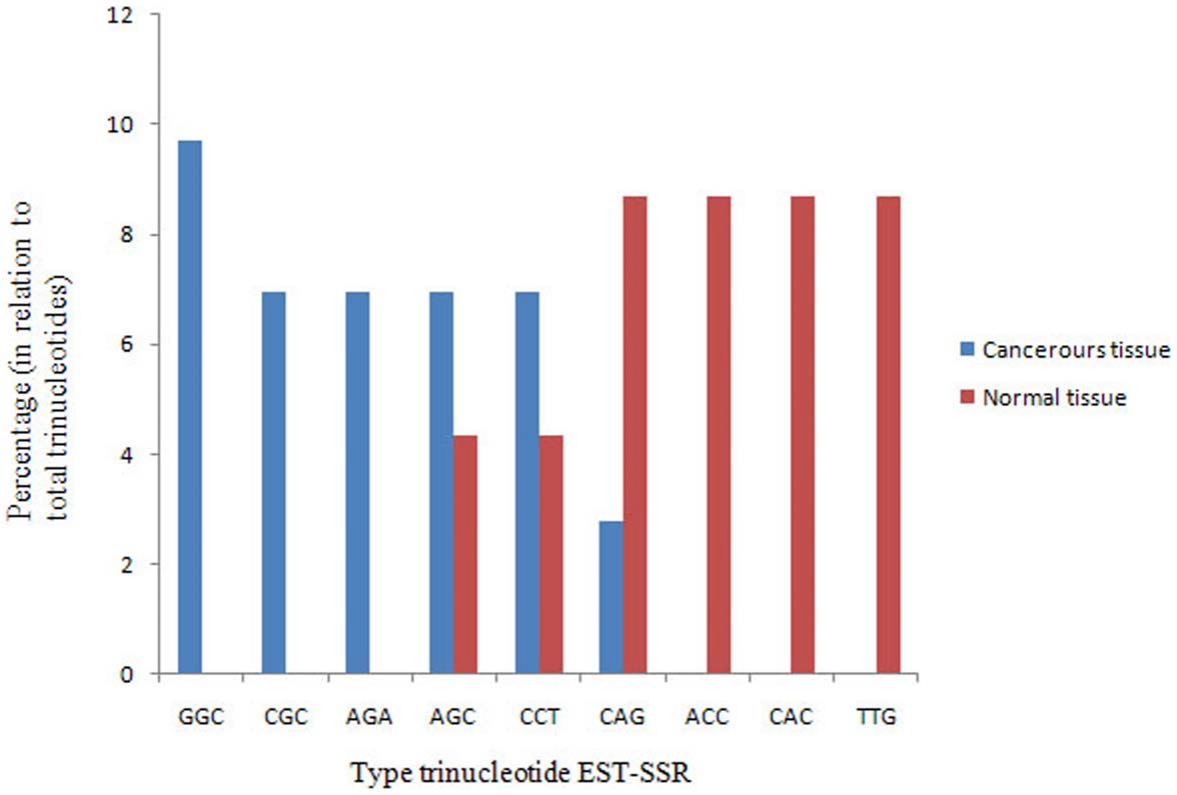
The distribution of different sequences of expressed trinucleotide SSRs in normal and cancerous lung tissues. A shift in trinucleotide EST-SSRs occurs when the tissue becomes tumorigenic, with decreases being observed in ACC, CAC, and TTG triplet tandem repeats and increases in GGC, CGC, and AGA instead.

**Figure 4:**
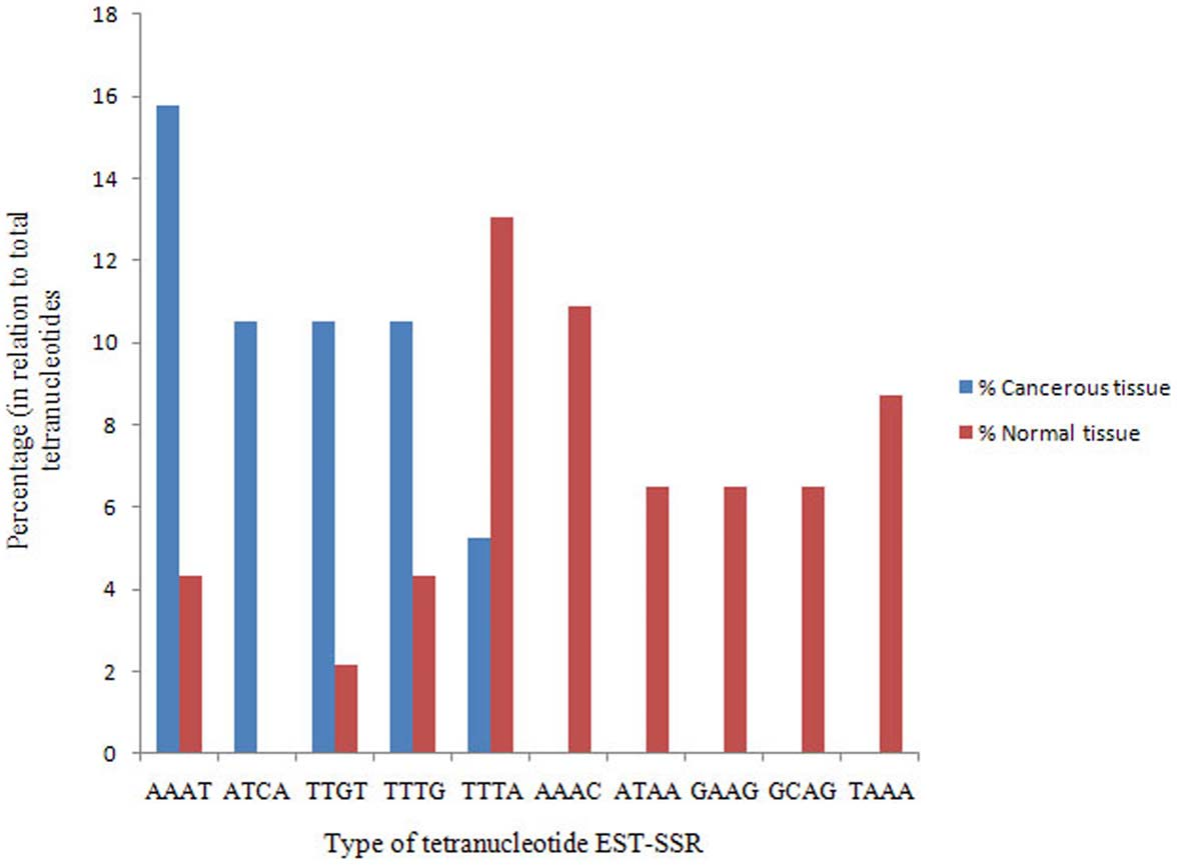
The distribution of tetranucleotide EST-SSRs in normal and cancerous lung tissues. Tetranucleotides, such as AAAC, ATCA, TTGT, GAAG, and TAAA, were differentially expressed between normal and cancerous tissues.

To shed light on the expressed SSR sequence modulations in each class of repeat units during lung cancer, the frequencies of the sequences within each class of repeat unit (dinucleotides, trinucleotides, tetranucleotides, pentanucleotides, or hexanucleotides) were compared between normal and cancerous tissues (Figures 2, 3, 4, Supporting Information S1).

**Figure 5:**
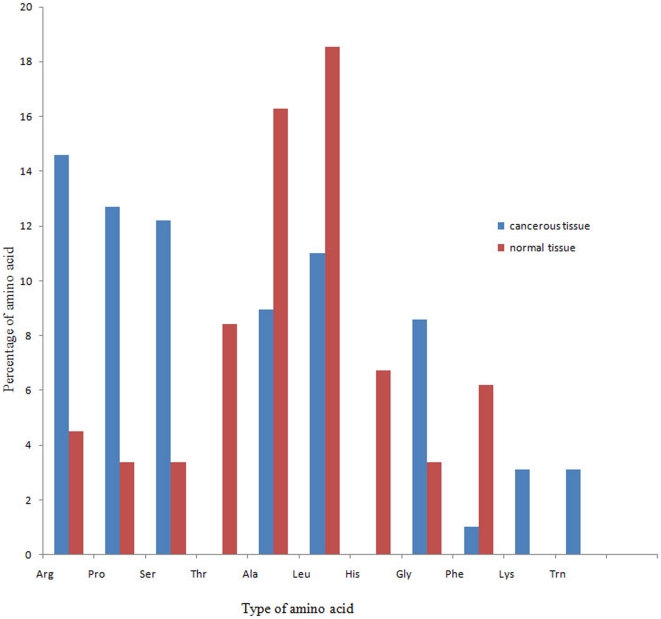
The percentages of different amino acids on ESTs containing trinucleotide tandem repeats in normal and cancerous tissues. Figure 5 highlights the high proportion of Arg, Pro, and Ser amino acids in cancerous lung tissue.

The types of dinucleotide sequences in cancerous and normal tissue are presented in Figure 2. Five dinucleotides, AC/GT, AG/CT, AT/TA, CA/TG, and GA/TC, were identified in both libraries, while GC/GC was solely detected in cancerous tissue (Figures 2, Supporting Information S1). Interestingly, the distribution of dinucleotide sequences was not similar between tissues. More than 40% of dinucleotides in the cancerous lung tissues were AT/TA-type. In contrast, this ratio decreased to 14.75% in normal tissue (Figures 2, Supporting Information S1). Thus, we suggest that the AT/TA and GC/GC frequencies can be employed to detect lung cancer.

For trinucleotides, 28 types of triplet repeats were found in cancerous tissue, while only 19 sequence-types were detected in normal tissue (Supporting Information S1). A differential distribution of trinucleotides in normal and cancer libraries was highly noticeable. While in normal tissue, triplet repeats were distributed with nearly equal frequencies, the distribution of triplet repeats was completely non-uniform in cancerous tissue. GGC and CGC were the more frequent expressed trinucleotide repeats in cancerous tissue (9.72% and 6.94%, respectively), while these SSRs were not expressed in the normal tissue. In contrast, ACC, CAC, and TTG, which were the dominant triplet tandem repeats in normal tissue, accounted for 26% of all expressed trinucleotides in normal tissue, rapidly disappear when the lung tissue becomes tumorigenic (Figure 3). Thus, monitoring the expression pattern of triplet repeats can be considered as an suitable way for lung cancer prediction and detection.

Figure 4 presents the tetranucleotide EST-SSR differences between normal and cancerous tissues. AAAC was highly prevalent in the normal library, whereas ATCA was highly prevalent in cancerous tissue. However, differences in the tetranucleotide SSR expression between cancer and normal were not as clear as trinucleotide EST-SSRs.

Pentanucleotides and hexanucleotides EST-SSRs are shown in Supporting Information S1. All five pentanucleotides in cancerous tissues had similar frequencies, while ATTCC showed the highest frequency in normal lung tissues (Supporting Information S1). Three hexanucleotides were found in the ESTs of lung cancer, including GCCCCA, CCTTGG, and CAACAG, while just one hexanucleotide (ATTTTT) was detected in normal ESTs (Supporting Information S1).

The number of EST-SSRs in each class of repeat units has been compared between cancerous and normal tissues and is presented in Table 2. Lung cancerous tissue statistically has a greater number of trinucleotide tandem repeats (P = 0.01). This finding confirms the probable role of trinucleotide EST-SSRs in cancer induction, which has been reported previously in other cancers [14], [15]. As presented in Table 2, the smaller tandem repeats (dinucleotides and trinucleotides) were more abundant in cancerous tissue than in normal tissue. In contrast, the frequencies of tetranucleotides and pentanucleotides (larger size tandem repeats) are higher in normal tissue.

**Table 2:** A comparison of the number of different EST-SSR types (dinucleotides, trinucleotides, tetranucleotides, and pentanucleotides) between normal and lung cancerous libraries by Paired T-test.

	Mean number in cancerous tissue (Mean ± SE)	Mean number in normal tissue(Mean ± SE)	P-Value*
Dinucleotides	22.50±8.00	20.33±5.37	NS
Trinucleotides	1,94±0,30	0.62±0.11	P = .01
Tetranucleotides	0.16±0.16	4. 00±0.51	P = 0.01
Pentanucleotides	0.30±0.13	1.15±0.27	P = 0.05
Hexanucleotides	1.75±0.85	0.25±0.25	NS

*NS: not significant, P = 0.05: significant at the 5% level, P = 0.01: significant at the 1% level.

### Correlation of expressed SSRs between lung cancerous and normal tissues

The correlation of expressed SSR sequences between normal and cancerous tissues in each class of tandem repeats (dinucleotides, trinucleotides, tetranucleotides, or pentanucleotides) is presented in Table 3. Interestingly, a negative correlation was found between expressions of trinucleotide-types between cancerous- and normal-tissue libraries (Table 3), while correlations of dinucleotides or tetranucleotides between normal and cancerous libraries were positive (Table 3). In fact, in line with the alteration of normal tissue to tumor, the expression profile of trinucleotide EST-SSRs appears to change. Expressed trinucleotide tandem repeats may be the best candidate for detecting and predicting cancerous lung tissue in future studies.

**Table 3:** A comparison of the correlation between the expressed SSR sequences in cancerous and normal lung tissues in generating different type of SSRs in each SSR class (the Pearson correlation test was used).

SSR class	Correlation between Cancerous and Normal libraries	P-value
Dinucleotides	+47.0%	Not significant
Trinucleotides	−32.4%	P = 0.05
Tetranucleotides	+77.5%	P = 0.1
Pentanucleotides	−46.0%	Not significant

### Virtual PCR

In the pooled cancerous library, 156 EST-SSRs had proper flanking regions for primer design. Consequently, 156 primers (69% of EST-SSRs) were designed for 227 EST-SSRs in cancerous tissues (Supporting Information S2). When virtual PCR was run with the SSR Locator software, 81 of the primers (52%) produced suitable fragments.

Based on proper flanking region, 113 primers were identified for 184 EST-SSRs (61% of EST-SSRs) in the normal library (Supporting Information S3), and 93 of these primers (82% of primers) produced SSR fragments during virtual PCR (Supporting Information S3). The primer sequences are presented in Supporting Information S2 and S3 and are useful in further laboratory studies on lung EST-SSRs.

### Functional annotation of EST-SSRs

To explore the functions of the EST sequences containing SSRs in both normal and cancerous tissues, BLASTX was used to search for EST-SSRs in the non-redundant protein (nr) databank of NCBI. A total of 117 out of 227 EST-SSRs in cancerous lung tissue and 55 out of 187 (30%) sequences in normal lung tissues had significant hits (Supporting Information S4 and Supporting Information S5).

A comparative functional annotation of EST-SSRs between normal and cancerous lung tissues is presented in Supporting Information S6. In addition, annotated proteins for trinucletotide EST-SSRs have been compared between normal and cancerous lung tissues in Supporting Information S7.

Many of the annotated EST-SSRs in cancerous tissue were related to chromodomain helicase DNA binding proteins, formin-binding proteins, and Chromobox protein homologues, and these genes did not express with SSRs in the normal library (Supporting Information S6). Chromodomain helicase DNA binding proteins solely produce from trinucletotide EST-SSRs in cancerous tissue (Supporting Information S7). In other words, SSRs in cancerous tissue prefer to join and target the nucleus proteins involved in heterochromatin formation, transcriptional activation, regulation of binding, and maintenance of heterochromatin integrity.

In particular, interference of SSRs with chromodomain helicase DNA binding proteins may result in the generation of defective genes in lung cancer. This finding is in agreement with the results of Li et al. (2004) regarding the effects of SSR distribution on chromatin organization and regulation of gene activity [13].

CCAAT/enhancer-binding protein α was another interesting target that was affected by SSRs solely in cancerous tissue; this protein has a well-documented role in cell proliferation. CCAAT-enhancer-binding proteins (or C/EBPs) are a family of transcription factors that interact with the CCAAT (cytidine-cytidine-adenosine-adenosine-thymidine) box motif, which is present in several gene promoters. C/EBPs are characterized by a highly conserved basic-leucine zipper (bZIP) domain at the C-terminus. This domain is involved in dimerization and DNA binding, as are other leucine zipper transcription factors. C/EBPs proteins are involved in different cellular responses, such as the control of cellular proliferation, growth and differentiation, metabolism, and immunology [31] [32].

The above findings open a new avenue in lung cancer studies and suggest that the SSRs may be both a marker for and also a cause of cancer induction. More studies in the future are needed in this field.

### Amino acid distribution of ESTs containing trinucleotide tandem repeats

Regarding the above-mentioned results on the importance of triplet repeats in lung cancer induction, the types of predicted amino acids and their distributions for ESTs with trinucleotide repeats were analyzed in normal and cancerous libraries. In keeping with the EST (mRNA) level, the expression pattern of EST-SSRs at the amino acid level was quite different.

The number of amino acid repeats was approximately 3 times higher in cancerous tissue than in normal tissue (582 versus 178 repeats, respectively, p = 0.01, Table 4). In addition, the type of expressed amino acids was notably different between cancerous and normal tissues (Table 4, Figure 5). Arg (14.60%), Pro (12.71%), Ser (12.19%), Leu (10.19%), and Ala (8.03%) were the most abundant amino acids in lung cancer tissues. In contrast, Leu (18.53%), Ala (16.23%), Thr (8.42%), Gln (8.42%) were the dominant amino acids in normal tissue. No Thr or His was found in the EST-SSRs of cancerous samples. On the other hand, Lys, Met, and Try were not found in normal tissues. The result of the amino acid study of EST-SSRs clearly shows that the induced instability of SSRs in cancerous tissue not only affect mRNA production, but also has a strong direct effect on the produced protein.

**Table 4:** A comparison of the amino acid distributions on EST-SSRs containing trinucleotide tandem repeats between cancerous and normal tissues.

Amino acid type	Number of amino acid loci		Number of amino acid repeats		Percentage of different type of amino acids repeats to the total number of repeats	
	Cancer	Normal	Cancer	Normal	Cancer	Normal
Ala	8	1	52	29	8.93%	16.29%
Arg	14	1	85	8	14.60%	4.49%
Asn	1	1	8	6	1.37%	3.37%
Asp	4	1	24	6	4.12%	3.37%
Cys	2	2	17	14	2.92%	7.86%
Gln	7	2	41	15	7.04%	8.42%
Glu	4	1	31	6	5.32%	3.37%
Gly	7	1	50	6	8.59%	3.37%
His	0	2	0	12	0.00%	6.74%
Ile	2	1	17	5	2.92%	2.80%
Leu	7	4	64	33	10.99%	18.53%
Lys	3	0	18	0	3.09%	0.00%
Me	1	0	6	0	1.03%	0.00%
Phe	1	2	6	11	1.03%	6.17%
Pro	12	1	74	6	12.71%	3.37%
Ser	10	1	71	6	12.19%	3.37%
Thr	0	2	0	15	0.00%	8.42%
Trn	3	0	18	0	3.09%	0.00%
Tyr	0	0	0	0	0.00%	0.00%
Val	0	0	0	0	0.00%	0.00%
Total	86	23	582	178		

Statistical comparison of amino acid distributions of EST-SSRs containing trinucleotide tandem repeats between cancerous and normal tissues						
			Cancer*	Normal*	Paired T-test**	
Number of amino acid loci			4.30±0.94	1.15±0.22	P = 0.01	
Number of amino acid repeats			29.10±6.21	8.90±2.02	P = 0.01	

*Means ± SE.

**P = 0.01: significant at the 1% level.

### EST-SSR distributions within cancerous tissues

**Figure 6:**
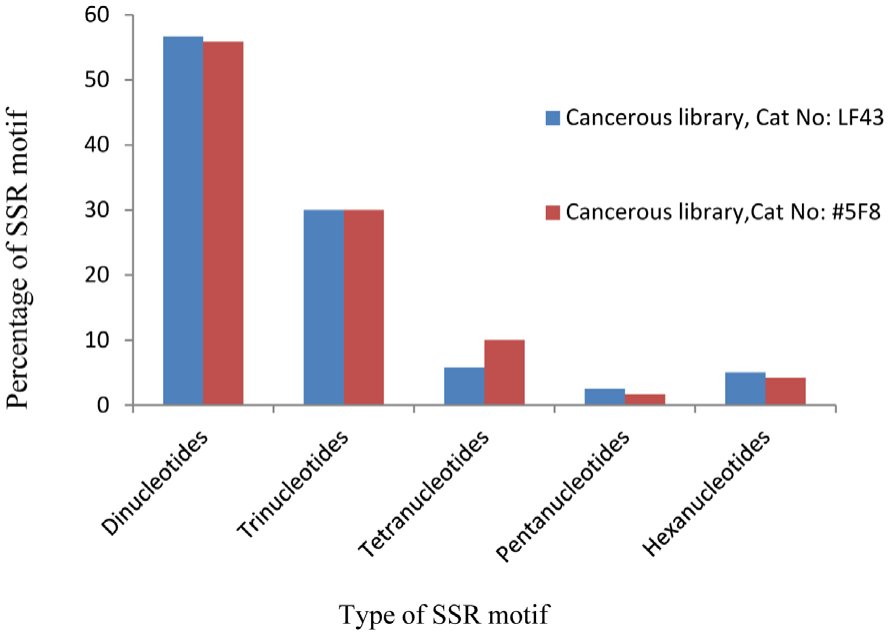
The distribution of EST-SSRs in normal and cancerous lung tissues. The percentages of dinucleotides, trinucleotides, tetranucleotides, pentanucleotides, and hexanucleotides have been compared between cancerous and normal tissues. EST-SSRs present similar distribution patterns between cancerous tissues.

Figure 6 presents the distribution of different classes of SSRs (dinucleotides, trinucleotides, tetranucleotides, pentanucleotides, and hexanucleotides) on ESTs between 2 different cancerous tissues. In addition, the different types of SSRs in each class were compared by Paired T-test (Supporting Information S7). As shown in Figure 6 and Supporting Information S8, there is no significant difference between the different classes of the 2 cancerous libraries, and EST-SSRs have similar expression patterns between cancerous tissues.

## Discussion

Lung cancer is the leading cause of cancer deaths in both women [27] and men [28] throughout the world. Most lung tumors are malignant, and only about 2% of those diagnosed with metastatic lung cancer are alive 5 years after the diagnosis [28]. Diagnosis of lung cancer in earlier stage sharply increases the survival rates to 49% for five years or longer.

One of the most prominent applications of molecular markers is the detection of diseases in early stages. However, no reliable maker has been introduced for the prediction of lung cancer. Microsatellites are the current method of choice because SSRs can be traced either in protein-coding or non-coding regions [33] with high mutability and may play a significant role in genome evolution [17]. EST-SSRs by following the behavior of SSRs in the coding part of genome, serve to monitor the modulation of microsatellites and also provide valuable information about the effects of SSRs on disease induction and functional alteration of genes during the disease.

In this study, we investigated SSR distribution in tumorigenic and normal lung tissues to search for new clues at molecular level of this cancer. To achieve this purpose, tumor libraries from lung cancer were compared with normal lung tissue in three steps: EST-SSR modulation, amino acid composition of translated EST-SSRs, and functional annotation of produced EST-SSRs. Analyzing a large number of ESTs in lung cancerous and normal tissues (24870) provided a useful estimate of genome modulation and alteration in lung cancer.

At the dinucleotide level, GC/GC was solely detected in cancerous tissue. GGC and CGC were the more frequently expressed trinucleotide repeats in cancerous tissue, while these SSRs were not expressed in normal tissue. In fact, tumorigenic lung ESTs were rich in GC content in comparison with normal ESTs. This observation is in agreement with previous reports of other cancers in human [2], animals [34], and even in plants [35]. The possible roles of GC content and repeat expansion in some human diseases have been previously highlighted [36].

The greatest difference between normal and cancerous tissues was observed in the expression of trinucleotides tandem repeats within all of studied repeats. The number of trinucleotides in lung cancer ESTs was 3 times higher than in normal ESTs (P = 0.05). In addition, the Pearson correlation test demonstrated that cancerous tissues generate different types of trinucleotides because negative and significant correlations (P = 0.05, Table 3) were found between cancer and normal tissues in terms of the expression of trinucleotide types. A differential distribution of trinucleotides in normal and cancer libraries was highly noticeable; while triplet repeats were distributed with nearly equal frequencies in normal tissue, the distribution of triplet repeats was non-uniform in cancerous tissue.

In keeping with the results of the present study, microsatellite instability has also been found in tumor samples of Turkish patients with breast cancer [37]. Microsatellite instability may reflect replication errors that are induced by defective mismatch repair genes, resulting in the appearance of novel, non-inherited alleles in tumor cells, and represents a specific pathway of tumor development. Interestingly, this developmental pathway is correlated with the clinicopathological features of breast cancer [3].

The possible structures of SSRs, particularly triplet repeats, which are involved in human diseases have been studied extensively [38], [39], [40], [41], [42], [43]. Various repeats have shown considerable potential to form alternative structures, such as (CTG)_n_, (CAG)_n_, (CCG)_n_, (CGG)_n_, (GAA)_n_, (TTC)_n_, (AGG)_n_, (CCT)_n_, (TGG)_n_ and (CCA)_s_ [44]. Therefore, tandem repeats may provide valuable clues regarding human diseases and provide new tools for disease identification, especially cancers.

The frequency of SSRs in EST-SSRs more accurately reflects the density of SSRs in the transcribed region of the genome. Because SSRs are inherently unstable, two models have been proposed to explain their generation and instability: DNA polymerase slippage (involving the transient dissociation of the replicating DNA strands) and unequal recombination followed by misaligned re-association. Those SSR repeats that can form alternative DNA conformations would be expected to be generated more frequently than others [45].

Translating the EST-SSRs to their corresponding amino acids provided some clues about differences and similarities between normal and cancerous tissues at the protein level. Highly significant differences (P = 0.01) were found between the type of amino acids and their distributions in normal and cancerous tissues (Table 4). Arg, Pro, Ser, Gly, Lys and Thr were the most abundant amino acids in lung-cancer tissues, while the frequencies of Leu, Cys, Phe, His and Thr were significantly higher in normal lung tissues (Table 4). The length of the amino acid tracts encoded by SSRs may affect the protein-protein interactions of transcription factors [14]. Microsatellites are known to participate in both gene and protein function [14]. These differences confirm the importance of SSRs, particularly triplet tandem repeats, in lung cancer.

In our previous work on breast cancer [46], we observed that the frequency of certain structural amino acid properties, such as Ile-Ile, can efficiently and precisely predict and discriminate malignant from benign breast-cancer cells. In similar investigations, structural amino acid attitudes has been efficiently predicted and modeled, including protein thermostability [47], [48], [49] and halostability [50], hyperaccumulation of a protein pump [51], or even the functional phylogeny of genes/proteins [52] using data mining methods. Regarding the abovementioned reports, the differential distribution of amino acids between cancerous and normal tissues provides a new avenue in lung cancer research to model and predict the cancer progression based on amino acid modulation.

Analyzing EST-SSRs instead of genomic SSRs provides the opportunity of following the behavior of a marker at the functional and protein level. Several EST-SSRs in cancerous tissue were related with chromodomain helicase DNA binding proteins and formin-binding proteins, and the expression of these proteins was not observed in normal library.

Alternative DNA conformations are one of the major causes of repeat expansion diseases in humans [45]. With respect to the function of Chromodomain helicase DNA binding proteins, Chromobox protein homologues, and CCAAT/enhancer-binding protein α in transcription, this study suggests valuable clues on how microsatellite instability, particularly in triplet tandem repeats, affects transcription, induces replication errors, and generates defective genes in lung cancer.

Repeat expansion diseases are a group of human genetic disorders caused by long and highly polymorphic tandem repeats. While the numbers of EST-SSRs in cancerous and normal tissues of lung were relatively similar (227 versus 184), the density of SSRs in cancerous tissues was significantly higher, probably due to changes in nucleotide sequences in abnormal tissues. Possible relationships between SSRs (especially trinucleotides) and hereditary and genetic diseases have been reported [2], [36], [53], [54], [55]. Nearly 30 hereditary disorders in humans result from an increase in the number of copies of simple repeats in genomic DNA. These DNA repeats seem to be predisposed to such expansion because they have unusual structural features that disrupt the cellular replication, repair and recombination machineries. The presence of expanded DNA repeats alters gene expression in human cells, leading to disease [54].

In conclusion, while the EST-SSR distribution was uniform between cancerous tissues, significant differences were found between the expression pattern of SSRs (particularly trinucleotides) and amino acid distributions in normal and tumorigenic tissues. These findings confirmed that the types and distributions of SSRs in normal tissues are different from those of unhealthy tissues in lung cancer; therefore, EST-SSRs may be suitable candidates for lung cancer studies. Functional analysis of EST-SSRs at the protein level led us to chromodomain helicase DNA binding protein and CCAAT/enhancer-binding protein α as a possible enzyme that may interrupt DNA replication and transcription in cancerous tissue. Based on the roles of SSRs with more than two nucleotides in cancer and other hereditary disorders and the significant differences found in the amino acid distributions in normal and cancerous lung tissues in this study, a possible relationship between SSR distributions and lung cancer was highlighted in this study.

The results of this study clearly show that contrary to previous assumptions, SSRs are not merely markers at the DNA level. More importantly, SSRs intentionally target and express with specific genes in cancer tissues. As shown in this research, the differential expression of SSRs in cancerous tissues affects amino acid and functional protein as well. These findings open a new avenue for finding important defective genes/proteins that have been tagged by SSRs, specifically in lung cancer.

Marker studies, up to now, focus mainly on marker, and little attention has been paid to the genes which are affected by markers. This study highlights the importance of investigating the genes which interact with markers and suggests following the behavior of marker-related genes in amino acid and protein level.

## Supporting Information

Supporting Information S1 Distribution of different EST-SSR sequences in normal and cancerous lung tissues, including dinucleotide, trinucleotide, tetranucleotide, pentanucleotide, and hexanucleotide tandem repeats.
(DOCX)

Supporting Information S2 Primer sequences for PCR of EST-SSRs in the cancerous lung library.
(XLS)

Supporting Information S3 Primer sequences for PCR of EST-SSRs in the normal lung library.
(XLS)

Supporting Information S4 Blastx result of EST-SSRs in cancerous lung tissue.
(XLS)

Supporting Information S5 Blastx result of EST-SSRs in normal lung tissue.
(XLS)

Supporting Information S6 Comparative functional annotation of EST-SSRs between normal and cancerous lung tissues. Similar proteins are in green.
(XLS)

Supporting Information S7 Comparative functional annotation of trinucletotide EST-SSRs between normal and cancerous lung tissues. Trinucleotide EST-SSRs are more abundant in the cancerous library than the normal library. Chromodomain helicase DNA binding proteins solely produce from trinucletotide EST-SSRs in cancerous tissue.
(XLS)

Supporting Information S8 Distribution of different EST-SSR sequences within cancerous tissues.
(DOCX)
