# Supplementary material for: Discovery of EST-SSRs in Lung Cancer: Tagged ESTs with SSRs Lead to Differential Amino Acid and Protein Expression Patterns in Cancerous Tissues
Source: PLoS One. 2011 Nov 4;6(11):e27118. doi: 10.1371/journal.pone.0027118 (PMC3208562; doi:10.1371/journal.pone.0027118)
Supplement: Supporting Information S1 — Distribution of different EST-SSR sequences in normal and cancerous lung tissues, including dinucleotide, trinucleotide, tetranucleotide, pentanucleotide, and hexanucleotide tandem repeats. (DOCX) [file pone.0027118.s001.docx]

**Supporting Information S1. Distribution of different EST-SSR sequences in normal and cancerous lung tissues, including dinucleotide, trinucleotide, tetranucleotide, pentanucleotide, and hexanucleotide tandem repeats.**

Percentage for each sequence was counted by dividing the number of that sequence to the total number of sequences in EST-SSR group.

|  |  | Cancerous tissue | |  | Normal tissue | |
| --- | --- | --- | --- | --- | --- | --- |
| Type of EST-SSR |  | Number of EST-SSR | Percentage of EST-SSR |  | Number of EST-SSR | Percentage of EST-SSR |
|  |  |  |  |  |  |  |
|  |  |  |  |  |  |  |
| Dinucleotides |  |  |  |  |  |  |
| AC/GT |  | 34 | 25.18% |  | 38 | 31.14% |
| AG/CT |  | 9 | 6.66% |  | 22 | 18.03% |
| AT/TA |  | 55 | 40.74% |  | 18 | 14.75% |
| CA/TG |  | 23 | 17.03% |  | 30 | 24.59% |
| GA/TC |  | 13 | 9.63% |  | 14 | 11.47% |
| GC/GC |  | 1 | 0.74% |  | 0 | 0.00% |
| Total Number |  | 135 |  |  | 122 |  |
|  |  |  |  |  |  |  |
| Trinucleotides |  |  |  |  |  |  |
| AAC |  | 0 | 0.00% |  | 1 | 4,35% |
| AAG |  | 3 | 4,16% |  | 0 | 0.00% |
| AAT |  | 1 | 1,38% |  | 0 | 0.00% |
| ACC |  | 0 | 0.00% |  | 2 | 8,69% |
| AGA |  | 5 | 6,94% |  | 0 | 0.00% |
| AGC |  | 5 | 6,94% |  | 1 | 4,35% |
| AGG |  | 2 | 2,78% |  | 1 | 4,35% |
| ATA |  | 1 | 1,39% |  | 0 | 0.00% |
| ATC |  | 1 | 1,39% |  | 0 | 0.00% |
| ATG |  | 1 | 1,39% |  | 0 | 0.00% |
| CAA |  | 3 | 4,17% |  | 0 | 0.00% |
| CAC |  | 0 | 0.00% |  | 2 | 8,69% |
| CAG |  | 2 | 2,78% |  | 2 | 8,69% |
| CCG |  | 1 | 1,39% |  | 0 | 0.00% |
| CCT |  | 5 | 6,94% |  | 1 | 4,35% |
| CGC |  | 5 | 6,94% |  | 0 | 0.00% |
| CGG |  | 2 | 2,78% |  | 0 | 0.00% |
| CTC |  | 1 | 1,39% |  | 0 | 0.00% |
| CTG |  | 4 | 5,55% |  | 1 | 4,35% |
| GAA |  | 1 | 1,39% |  | 1 | 4,35% |
| GAG |  | 3 | 4,17% |  | 0 | 0.00% |
| GAT |  | 4 | 5,55% |  | 1 | 4,35% |
| GCA |  | 1 | 1,39% |  | 0 | 0.00% |
| GCC |  | 3 | 4,17% |  | 0 | 0.00% |
| GCT |  | 0 | 0.00% |  | 1 | 4,35% |
| GGA |  | 0 | 0.00% |  | 1 | 4,35% |
| GGC |  | 7 | 9,72% |  | 0 | 0.00% |
| TAA |  | 0 | 0.00% |  | 1 | 4,35% |
| TCC |  | 1 | 1,39% |  | 0 | 0.00% |
| TCT |  | 4 | 5,55% |  | 0 | 0.00% |
| TGA |  | 0 | 0.00% |  | 1 | 4,35% |
| TGC |  | 2 | 2,78% |  | 1 | 4,35% |
| TGG |  | 1 | 1,39% |  | 0 | 0.00% |
| TGT |  | 0 | 0.00% |  | 1 | 4,35% |
| TTA |  | 2 | 2,78% |  | 1 | 4,35% |
| TTC |  | 1 | 1,39% |  | 1 | 4,35% |
| TTG |  | 0 | 0.00% |  | 2 | 8,69% |
| Total Number |  | 72 |  |  | 23 |  |
|  |  |  |  |  |  |  |
| Tetranucleotides |  |  |  |  |  |  |
| AAAC |  | 0 | 0.00% |  | 5 | 10,87% |
| AAAT |  | 3 | 15,79% |  | 2 | 4,35% |
| AGAT |  | 0 | 0.00% |  | 1 | 2,17% |
| AGGA |  | 0 | 0.00% |  | 2 | 4,35% |
| ATAA |  | 0 | 0.00% |  | 3 | 6,52% |
| ATAG |  | 0 | 0.00% |  | 1 | 2,17% |
| ATCA |  | 2 | 10,53% |  | 0 | 0.00% |
| CTGC |  | 0 | 0.00% |  | 1 | 2,17% |
| CTTT |  | 0 | 0.00% |  | 1 | 2,17% |
| GAAG |  | 0 | 0.00% |  | 3 | 6,52% |
| GAGC |  | 1 | 5,26% |  | 0 | 0.00% |
| GATG |  | 1 | 5,26% |  | 0 | 0.00% |
| GCAG |  | 1 | 5,26% |  | 0 | 0.00% |
| GCAG |  | 0 | 0.00% |  | 3 | 6,52% |
| GCGG |  | 1 | 5,26% |  | 0 | 0.00% |
| GCTG |  | 0 | 0.00% |  | 1 | 2,17% |
| GGTT |  | 0 | 0.00% |  | 1 | 2,17% |
| GTTT |  | 0 | 0.00% |  | 1 | 2,17% |
| TAAA |  | 0 | 0.00% |  | 4 | 8,69% |
| TAGA |  | 1 | 5,26% |  | 0 | 0.00% |
| TATT |  | 0 | 0.00% |  | 2 | 4,35% |
| TCAC |  | 1 | 5,26% |  | 0 | 0.00% |
| TCCT |  | 1 | 5,26% |  | 0 | 0.00% |
| TCTT |  | 0 | 0.00% |  | 2 | 4,35% |
| TGAT |  | 1 | 5.26% |  | 0 | 0.00% |
| TTCC |  | 0 | 0.00% |  | 1 | 2,17% |
| TGTT |  | 1 | 5,26% |  | 2 | 4.35% |
| TTGT |  | 2 | 10,53% |  | 1 | 2,17% |
| TTTA |  | 1 | 5,26% |  | 6 | 13,04% |
| TTTC |  | 0 | 0.00% |  | 2 | 4,35% |
| TTTG |  | 2 | 10,53% |  | 2 | 4.35% |
| Total Number |  | 19 |  |  | 46 |  |
|  |  |  |  |  |  |  |
| Pentanucleotides |  |  |  |  |  |  |
| AAACA |  | 1 | 25.00% |  | 0 | 0.00% |
| AATAA |  | 0 | 0.00% |  | 2 | 13,33% |
| AGAGA |  | 0 | 0.00% |  | 1 | 6,66% |
| ATTCC |  | 0 | 0.00% |  | 4 | 26,66% |
| CAAAA |  | 1 | 25.00% |  | 1 | 6,66% |
| CATTC |  | 0 | 0.00% |  | 1 | 6,66% |
| GAATG |  | 0 | 0.00% |  | 1 | 6,66% |
| GGAAT |  | 0 | 0.00% |  | 1 | 6,66% |
| GGGCT |  | 1 | 25.00% |  | 0 | 0.00% |
| TCCAT |  | 0 | 0.00% |  | 1 | 6,66% |
| TCCCT |  | 0 | 0.00% |  | 1 | 6,66% |
| TGGAA |  | 0 | 0.00% |  | 1 | 6,66% |
| TGTTT |  | 1 | 25.00% |  | 1 | 6,66% |
| Total Number |  | 4 |  |  | 15 |  |
|  |  |  |  |  |  |  |
| Hexanucleotides |  |  |  |  |  |  |
| GCCCCA |  | 4 | 57,14% |  | 0 | 0.00% |
| CCTTGG |  | 2 | 28,57% |  | 0 | 0.00% |
| CAACAG |  | 1 | 14,28% |  | 0 | 0.00% |
| ATTTTT |  | 0 | 0.00% |  | 1 | 100.00% |
| Total Number |  | 7 |  |  | 1 |  |
