# Supplementary material for: Discovery of EST-SSRs in Lung Cancer: Tagged ESTs with SSRs Lead to Differential Amino Acid and Protein Expression Patterns in Cancerous Tissues
Source: PLoS One. 2011 Nov 4;6(11):e27118. doi: 10.1371/journal.pone.0027118 (PMC3208562; doi:10.1371/journal.pone.0027118)
Supplement: Supporting Information S8 — Distribution of different EST-SSR sequences within cancerous tissues. (DOCX) [file pone.0027118.s008.docx]

**Supporting Information S8. Distribution of different sequences of EST-SSRs within cancerous tissues.**

Two EST libraries of [malignant](http://en.wikipedia.org/wiki/Malignant) lung [tumor](http://en.wikipedia.org/wiki/Tumor) were compared; undifferentiated large cell carcinoma library containing 6556 ESTs (Cat No: #5F8) and poorly differentiated squamous cell carcinoma containing 6662 ESTs (Cat No: LF43) were compared by Paired T-test. Paired T-test allows that the effect of different types of SSRs in each class of EST-SSR to be considered.

| Class of EST-SSR | Comparison of EST-SSR distribution between cancerous tissues by Paired T-test between different types of EST-SSRs | | | | | |  |
| --- | --- | --- | --- | --- | --- | --- | --- |
|  |  | mean ± SE mean in LF43 cancerous  library |  | mean ± SE mean in #5F8 cancerous library |  | P-value | |
| Dinucleotides |  | 6.18 ± 2.21 |  | 6.09 ± 1.42 |  | Not significant | |
| Trinucleotides |  | 1.333 ± 0.239 |  | 1.333 ± 0.272 |  | Not significant | |
| Tetranucleotides |  | 0.538 ± 0.144 |  | 0.923± 0.137 |  | Not significant | |
| Pentanucleotides |  | 0.600 ± 0.245 |  | 0.400 ± 0.245 |  | Not significant | |
| Hexanucleotides |  | 2.000 ± 0.577 |  | 1.000 ± 0.577 |  | Not significant | |
